# Recent progress in the development of nanomaterials targeting multiple cancer metabolic pathways: a review of mechanistic approaches for cancer treatment

**DOI:** 10.1080/10717544.2022.2144541

**Published:** 2023-01-03

**Authors:** Ling Zhang, Bing-Zhong Zhai, Yue-Jin Wu, Yin Wang

**Affiliations:** a Reproductive Medicine Center, Department of Reproductive Endocrinology, Zhejiang Provincial People’s Hospital, Affiliated People’s Hospital, Hangzhou Medical College, Hangzhou, Zhejiang, 310014, China; b Hangzhou Municipal Center for Disease Control and Prevention, Hangzhou, Zhejiang, 310021, China; c Institute of Food Science and Engineering, Hangzhou Medical College, Hangzhou, Zhejiang, 310013, China

**Keywords:** Cancer metabolisms, nanoparticles, anticancer drugs, nanomedicine, drug delivery

## Abstract

Cancer is a very heterogeneous disease, and uncontrolled cell division is the main characteristic of cancer. Cancerous cells need a high nutrition intake to enable aberrant growth and survival. To do so, cancer cells modify metabolic pathways to produce energy and anabolic precursors and preserve redox balance. Due to the importance of metabolic pathways in tumor growth and malignant transformation, metabolic pathways have also been given promising perspectives for cancer treatment, providing more effective treatment strategies, and target-specific with minimum side effects. Metabolism-based therapeutic nanomaterials for targeted cancer treatment are a promising option. Numerous types of nanoparticles (NPs) are employed in the research and analysis of various cancer therapies. The current review focuses on cutting-edge strategies and current cancer therapy methods based on nanomaterials that target various cancer metabolisms. Additionally, it highlighted the primacy of NPs-based cancer therapies over traditional ones, the challenges, and the future potential.

## 1. Introduction

Cancer is reported as the major cause of premature death in China and other developed nations (Bray et al., 2021). GLOBOCAN 2020 reported that there were 19,292,789 cases of cancer and 9,958,133 deaths from cancer worldwide in 2020 (Sung et al., 2021). Since 2000, the prevalence and mortality of cancer in China have risen gradually (Wei et al., 2020). Cancer cells are distinguished by their uncontrolled proliferation, transformation, and migration to other parts of the body, as well as their propensity to harm normal cells (Suresh, 2007). Cancer cells acquire atypical metabolic pathways to get energy and raw materials for achieving the energy requirements for vigorous cell growth and migration. The reason cancer cells acquire energy via atypical metabolic pathways is that their metabolism is more vigorous than that of normal cells (Zanotelli et al., 2021). Multiple carcinogenic signaling pathways regulate three primary metabolic pathways in cancer cells. These metabolic pathways include lipid, amino acid, and glucose metabolism (Dzobo et al., 2020). The distinctive metabolism of cancer cells implies that changes at the metabolic level are essential for the development and genesis of cancer cells.

Despite all the advances in medicine, chemotherapy and other traditional cancer treatments have not been completely successful due to certain factors associated with cancer. Cancers usually show resistance to therapeutic drugs currently used in cancer therapies (Dagogo-Jack and Shaw, 2018). Most chemotherapeutic drugs kill both healthy and cancerous cells in the body. Therefore, administering these chemotherapeutic drugs might result in serious adverse effects and occasionally even result in a patient’s death (Marin et al., 2016). Some other limitations associated with conventional therapies include less permeability and poor biocompatibility, and the major drawback is the poor specificity for targeting cancerous sites. For example, in radiotherapy, no cell specificity leads to serious adverse effects in cancer patients. Another concern is drug resistance, in which anticancer drugs occasionally fail to act entirely. Recurrence and metastasis are two additional major challenges in cancer treatment encountered by cancer therapists. Some cancer cells evade treatment and get a chance to reoccur in patients (Song et al., 2020). In metastasis, when the tumor spreads to nearby tissues and possibly the entire body, it becomes challenging to treat the malignancy.

**Figure 1.**
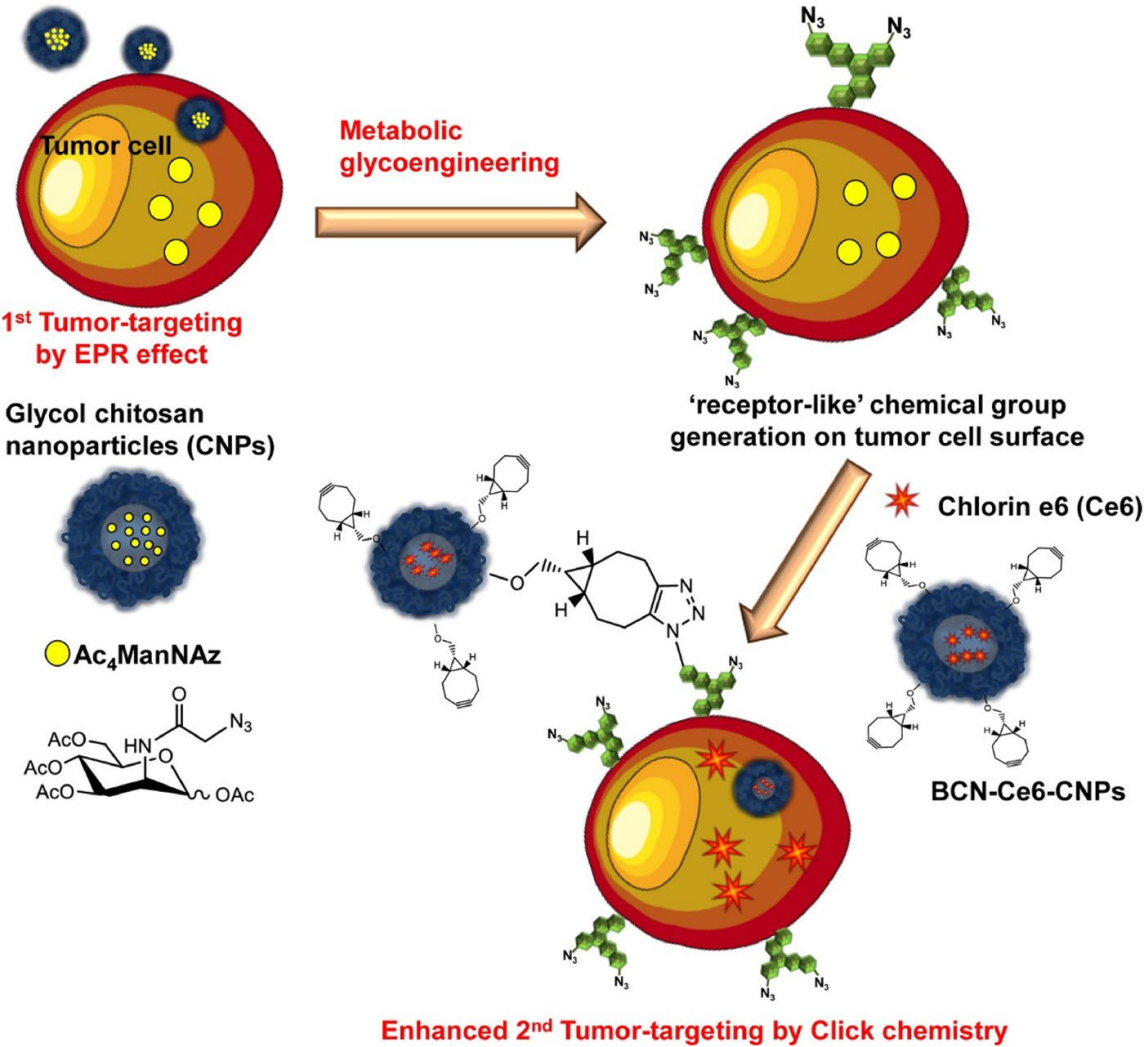
Schematic illustration of the two-step in vivo tumor-targeting strategy for nanoparticles via metabolic glycoengineering and click chemistry. Reproduced with permission from ACS 2014 (Lee et al., 2014).

Metabolic chemotherapy has been used successfully to treat cancer for decades, proving the existence of a therapeutic window for targeting malignant metabolism (Luengo et al., 2017). The synthesis of deoxynucleotide and nucleotide is the only metabolic pathway that has been extensively researched and successfully addressed in cancer therapy (Wilson et al., 2014; Mathews, 2015). However, discoveries and the effectiveness level of current anticancer treatments have shifted the focus of research onto other critical metabolic pathways. For example, there has been renewed interest in targeting polyamine metabolism as an anticancer therapy because of our improved understanding of polyamine metabolism, molecular activities, and their changes in cancer (Casero et al., 2018). A better understanding of the connection amongst cancer-driving pathways such as polyamine metabolism, RAS pathways, WNT signaling, and PTEN–PI3K–mTOR complex 1 (mTORC1) would lead to the existence of promising combination therapy. In light of recent discoveries about the molecular pathways connecting carcinogenesis and dysregulated polyamine catabolism provide additional cancer preventive methods for persons at risk (Amin et al., 2021). Furthermore, polyamine-blocking therapy, which involves inhibiting both polyamine transport and polyamine production, can be more successful than treatments based on polyamine reduction alone (Alexander et al., 2020). As a result of recent discoveries on the metabolic specificities of malignancies, novel treatment techniques have been developed to take advantage of metabolic disorders; some of these are now being tested in preclinical models and clinical trials (Raez et al., 2013; Ott et al., 2013). The use of nanoparticles is a novel therapeutic approach for treating cancer. It has attracted the interest of the cancer research community and is paving the way for new approaches to the diagnosis, characterization, and therapy of the disease because of its distinctive features (Valenzuela et al., 2021). This review focuses on cutting-edge cancer treatment approaches and cutting-edge nanomaterial tactics that target distinct cancer metabolisms. Additionally, it emphasized the difficulties and possibilities of NPs-based cancer therapy as opposed to conventional ones (Figure 1).

## 2. Metabolic pathways associated with cancer progression

Tumor suppressor loss and oncogene activation are crucial factors in the promotion of cancer cell metabolism reprogramming (Jones and Thompson, 2009). This change results in an improvement in the absorption of nutrients that are necessary to fuel biosynthesis pathways. Lack of nutrients affects solid tumors. Cancer cells adapt metabolic flexibility to help with the sustainability of growth and survival to get around this restriction. It is believed that the reprogramming of metabolic pathways is one of the primary characteristics of cancer (Nagao et al., 2019). That is why, over the past ten years, it has been the primary focus of cancer research. When nutrients are abundant, cancer signaling pathways ingest more nutrients to speed up the incorporation of carbon into different macromolecules like proteins, lipids, and nucleic acids (Spinelli and Haigis, 2018). These cellular activities support cell growth and proliferation.

**Figure 2.**
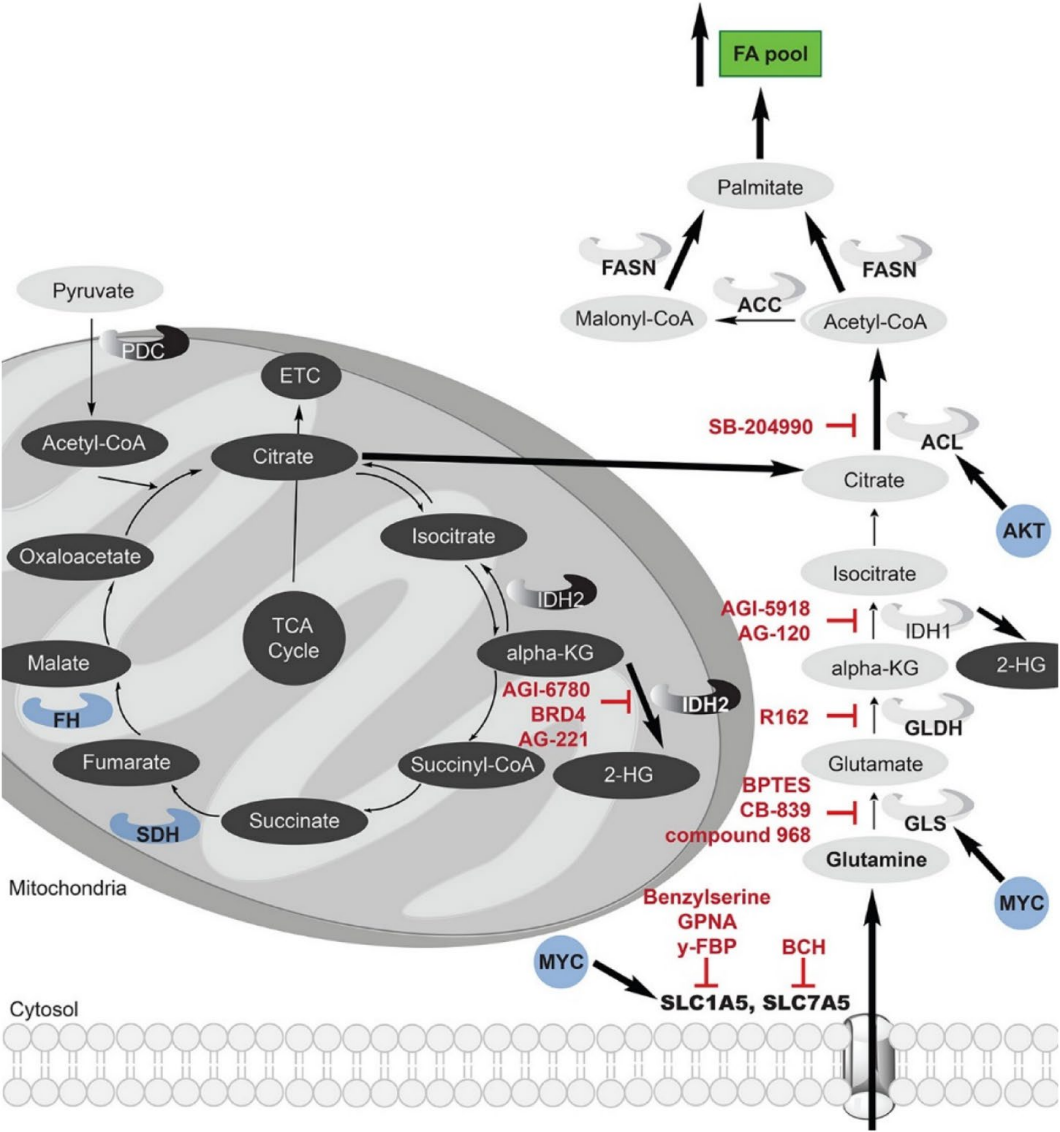
The tricarboxylic acid (TCA) cycle and the glutaminolysis pathway interact in cancer, and both are frequently dysregulated. The metabolism of glutamine is essential for the survival and growth of numerous types of cancer cells. Increased expression of the oncogene MYC results in an increase in glutamine absorption and glutaminolysis in cancer cells. Both the TCA cycle and glutaminolysis contribute to an increase in citrate and acetyl-CoA, which ultimately results in a rise in the fatty acid pool. Reactions that are upregulated in cancer cells are shown by thicker black arrows, and upregulated enzymes are highlighted in bold font. Red indicates current clinical or preclinical cancer medicines that target enzymes related to lipogenesis, the TCA cycle, or glutaminolysis. Cancer cell oncogenes that stimulate lipogenesis, the TCA cycle, or glutaminolysis are denoted in blue. Reproduce with permission from ACS 2018 (Counihan et al., 2018).

Mutations frequently reprogram the metabolism of glutamine and glucose in oncogenic pathways. The mutations are apparent in Myc proteins, tumor protein p53, the Ras-related oncogenes, the signaling pathways of PI3 kinase (1I3K), upstream kinase, liver kinase B1-Activated protein kinase (LKB1-AMP), and AMPK kinase (Hao et al., 2016; Wieman et al., 2007). Oncogenic Ras is involved in the stimulation of both glucose uptake via the uptake of glucose by anabolic pathways and the enhanced activity of Glucose transporter 1(GLUT1) (Gaglio et al., 2011). Ras plays a critical part in the control of glutamine metabolism by directing glutamine carbon in the direction of pathways that promote cell growth and survival. Increased c-Myc expression contributes to the manifestation of numerous metabolic effects, such as increased glycolysis, increased glutamine catabolism, increased mitochondrial biogenesis, and increased glutamine metabolism, which results in the assimilation of biomass (Miller et al., 2012; Dang, 2010). Multiple pathways converging on glutamine and glucose show that these nutrients are abundant and fed into the central metabolism. Additionally, the two nitrogen atoms in glutamine are used to create the amino acids, hexosamines, and nucleotides necessary for growth. In essence, cancer research shows that cancer cells have much more complex metabolic needs and that multiple pathways support the glutamine- and glucose-dependent production of biomass in cancer cells (Kamphorst et al., 2015) (Figure 2).

Despite the low blood pressure within the tumors, lymphatic insufficiency and blood vessel leaks play a role in making blood serum or its albumin protein accessible to cancer cells. Macropinocytosis occurs more frequently in Ras-driven tumors, which makes it easier for them to internalize extracellular proteins (ECPs) (Finicle et al., 2018). Additionally, it has been noted that inhibition of the mTORC1 signaling pathway causes cancer cells to become dependent on extracellular macromolecules rather than amino acids in the event of nutrient deprivation. In an experiment, AMPK activation and mTORC1 suppression in a state of glucose and amino acid starvation facilitate tumor proliferation by scavenging cell debris in Phosphatase and Tensin Homolog (PTEN) deficient prostate cancer cell lines and K-Ras-driven pancreatic tumors (Kim et al., 2018). Amino acids from cell debris participate in the building of cell biomass. Furthermore, when starved of glucose and glutamine, pancreatic ductal adenocarcinoma (PDAC) can internalize collagen I and IV. To produce proline, PDAC cells break down the internalized collagen in the lysosomes (Olivares et al., 2017). Extracellular signal-regulated kinase pathways that result in the production of ATP and, ultimately, cell survival feed proline into the tricarboxylic acid cycle. In vitro studies reveal that cancer cells utilize albumin as an alternative source of nutrients (Recouvreux and Commisso, 2017). An in vivo study shows that PDAC cells can take in albumin and use the amino acids it contains in other metabolic pathways, which normal cells can’t do (Lambies and Commisso, 2022).

Extracellular lipids can also be used by cancer cells to grow and survive in hypoxic or nutrient-limited environments (Menendez et al., 2004). Cancer cells require extracellular lipids and fatty acids for membrane reproduction and proliferation. Under normal circumstances, with easy access to oxygen, cancer cells produce non-essential fatty acids. Ras-driven cancers or hypoxic conditions, on the other hand, rely on fatty acids that are scavenged from the tumor microenvironment. Ras-driven cancer cells can internalize lipids with just one fatty acid tail to meet their lipid needs (Nguyen et al., 2017). In the state of starvation, the lipid droplet content of cancer cells also declines. PTEN-deficient prostate cancer cells are dependent on the lipids extracted from cell debris (Kim et al., 2018). Invasive breast tumors also show a higher proliferation and migration rate in co-culture with obese adipocytes. Adipocyte-derived fatty acids induce adipose triglyceride lipase (AGTL)-mediated lipolysis and fatty acid oxidation in the mitochondria when they are transferred to invasive breast cancer cells (Strong et al., 2015).

The function of acetate and acetate-metabolizing enzymes in cancer cells has recently been demonstrated (Schug et al., 2016). Both substrates could be oxidized when 13 C-glucose and 13 C-acetate were infused into mice with orthotopic glioblastomas. However, in plasma, the tumors oxidized more quickly than the nearby normal brain tissues did. Acetate oxidation has been seen in metastatic brain tumors, suggesting that this pathway is a common characteristic of tumors in the brain (Mashimo et al., 2014). Infusion of a similar mixture of 13 C-glucose and 13 C-acetate in human patients also revealed extensive acetate oxidation in gliomas and brain metastases. An Acyl-CoA Synthetase Short Chain 2 (ACSS2) deficient mouse model was used to examine the role of this enzyme in tumor growth (Huang et al., 2018). These embryonic fibroblasts lacking ACSS2 are unable to use exogenous acetate for lipogenesis and histone acetylation. Furthermore, ACSS2 knockout in two hepatocellular carcinoma models lessens the tumor burden (Huang et al., 2018; Bidkhori et al., 2018). According to the selective ACSS2 inhibitors hypothesis, targeting acetate metabolism may have a potent therapeutic effect in some types of cancer (Olson et al., 2016). Given the importance of the aforementioned cancer metabolic pathways, nanoparticles can be used to target specific metabolic sections in these pathways to identify potential cancer therapeutic strategies.

## 3. Nanoparticles-based cancer treatment exceeding conventional treatment

**Figure 3.**
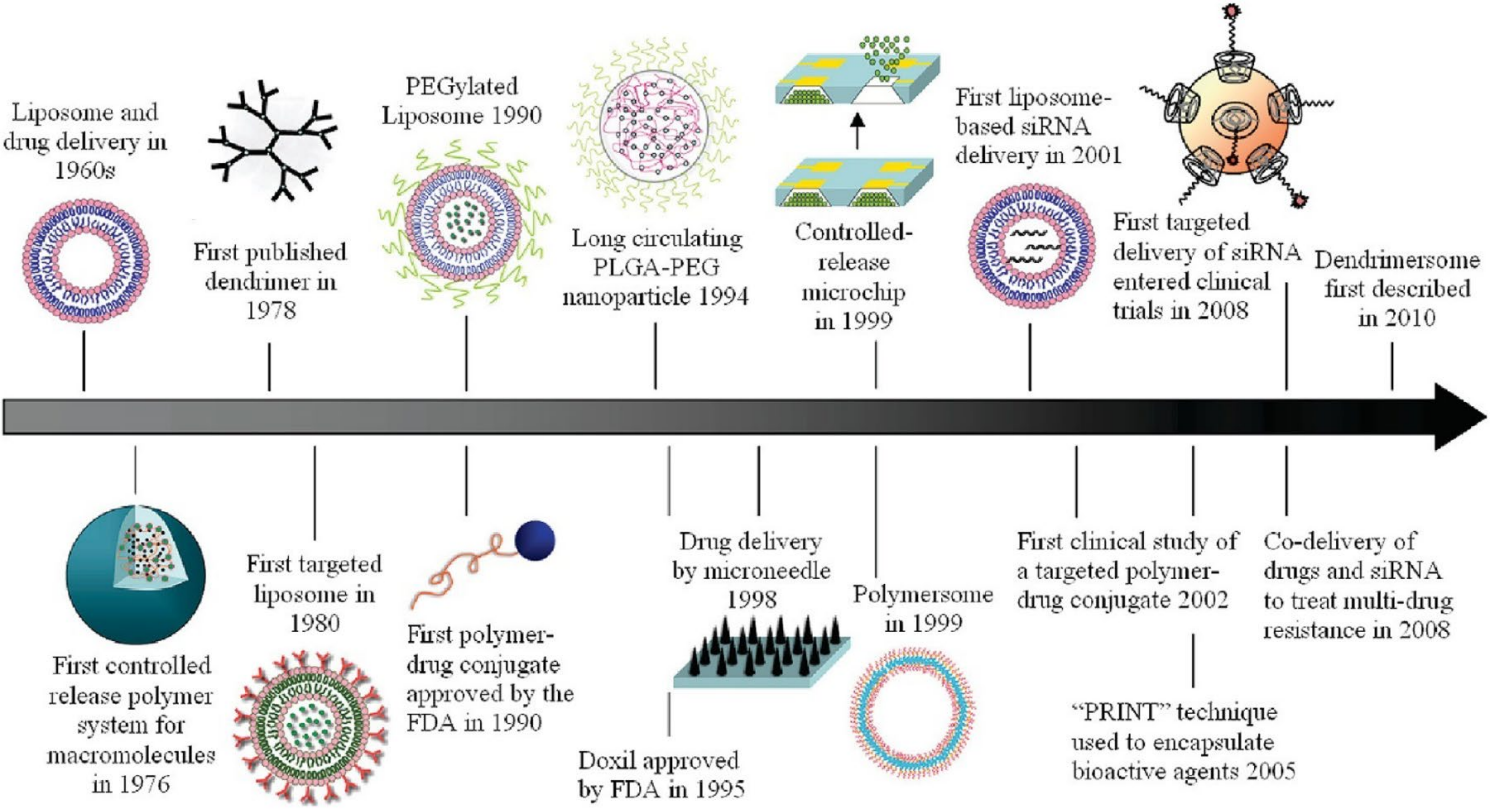
A historical look at the use of nanotechnology in the development of drug delivery systems, with a focus on a few key innovations. Reproduced with permission from ACS 2010 (Shi et al., 2010).

The targeted delivery of drugs into the cancer metabolic pathways is the significant advantage of nanoparticles (NPs). Targeted drug delivery in cancer has been the subject of extensive research and advancement. Either active targeting or passive targeting is used to achieve it (Ertas et al., 2021). Enhanced retention time and permeability are the properties of passive targeting, while in active targeting, NPs are conjugated with antibodies, peptides, aptamers, and other small molecules (Yoo et al., 2019). Drug delivery using targeted NPs reduces toxicity in healthy cells, stops drug degradation, and improves solubility, half-life, and drug loading capacity. In contrast to conventional chemical cancer treatment methods, NPs-based cancer treatment maintains better specificity, biocompatibility, less cytotoxicity, prolonged half-life, controlled drug release, and high loading capacity of drugs (Tang et al., 2021). Anticancer drugs are less likely to encounter resistance by the target cells when they are encapsulated with NPs; for example, the anticancer drug Metformin conjugated with NPs is more effective than using Met alone to treat cancer (Wei et al., 2020). Moreover, target-specific NPs have the potential to inhibit the genes involved in the control of metastasis and can be used to regulate those genes as well. Mucoadhesive NPs delivered intravenously in combination with Lysine Demethylase 6 A (KDM6A-mRNA) may prevent bladder cancer metastases (Kong et al., 2022). Following nanotechnology advancements, various nanotherapeutic drugs have been commercialized and marketed, and more have entered the clinical stage since 2010. The development of nanotherapeutic drugs has allowed for the possibility of drug combination therapy and the inhibition of drug resistance mechanisms, both of which are important advances in the field of drug delivery systems and anti-tumor multidrug resistance (MDR) (Palazzolo et al., 2018). Examples include Genexol PM®, a novel nanoformulated paclitaxel, and sterile lyophilized polymeric micellar formulation without CrEL. The maximum tolerated dose (MTD) for Genexol PM® was found to be three times higher in nude mice. Furthermore, the biodistribution showed concentrations that were two- to three-times higher in various tissues, particularly in cancer cells, than in healthy ones. It has been approved in South Korea to treat MBC. In the US, it is still being tested in phase II clinical trials for the treatment of pancreatic cancer (Lee et al., 2008). The solid-lipid based nanoparticles of the photo sensitizer drug verteporfin were developed specifically for the treatment of ovarian cancer. The developed nanosystem was efficiently internalized into the tumor environment following laser irradiation, leading to significant suppression of tumor cell viability. Note that free drug administration at a dose of 2 mg/kg resulted in adverse effects and toxicity-associated death, but drug-loaded solid - lipid nanoparticles resolved this issue and showed no toxicity after intravenous injection of the nanoformulation at a dose of 8 mg/kg (Michy et al., 2019) (Figure 3).

## 4. Application of NPs in cancer therapy

NPs with sizes ranging from 1-100 nm are used in the medical field. These NPs are used in manufacturing, designing, and developing therapeutic drugs and drug delivery systems (Ion et al., 2021). NPs set themselves apart from conventional macromolecules due to their distinct optical, magnetic, and electrical properties that emerge at the nanoscale. The nanoparticles’ typical properties include a high surface-to-volume ratio, super magnetic behavior, enhanced electrical conductivity, and distinctive fluorescence properties (Baig et al., 2021). NPs have the potential to deliver drugs with controlled release and exhibit enhanced biocompatibility and increased permeability. Due to their high surface-to-volume ratio, NPs can increase the specificity of drugs used in targeted therapy, increasing treatment effectiveness while lowering toxicity to healthy cells (Nitheesh et al., 2021). Different types of materials, including lipids, metal particles, polymers, and others, are used in the structure of NPs used for drug delivery. NPs can be prepared in a variety of sizes and forms depending on their structural makeup. Nanocarrier-based drug delivery systems have made their way into the international pharmaceutical market. Their use in drug delivery systems is expanding every day.

**Figure 4.**
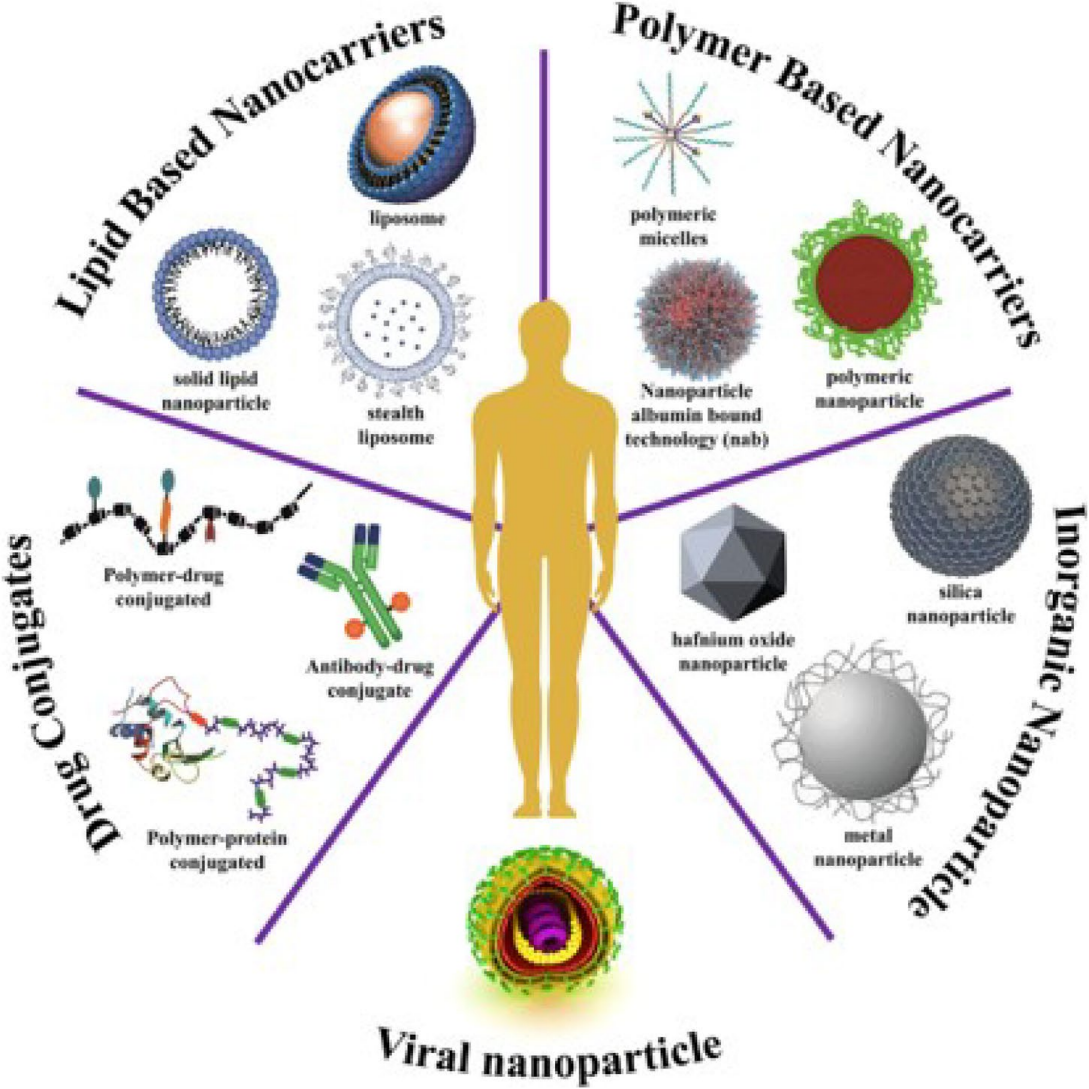
A synopsis of currently available nanomedicines for the treatment of cancer. Clinical studies are looking into a wide range of platforms as nanocarriers, such as drug-conjugated NPs, viral NPs, inorganic NPs, polymer-based NPs, and lipid-based NPs. Reproduced with permission from reference (Aghebati-Maleki et al., 2020).

NPs can be used for the diagnosis and treatment of cancer due to their special properties. Self-assembly of DNA nanostructures using nitrogen-enriched carbon dots (NCDs) has great promise for bioimaging and anticancer therapy because of its high photoluminescence and photodynamic properties (Wu et al., 2020). Superparamagnetic iron oxide particles (SPION) could be used in hyperthermia treatment where their high targeting specificity, small size, controlled release, and immune evasion capability make them effective for cancer treatment (Kianfar, 2021). Some nanoparticles can also be used in photothermal therapy (PTT) and photodynamic therapy (PDT) cancer treatment techniques due to their unique fluorescence characteristics. PDT and PTT are two methods of treatment associated with optical interference. Oxygenic hybrid ruthenium sulfide nanoclusters (RuSx NCs) with varying molar ratios of Ru to S exhibited a significant photothermal effect for the PTT of tumors of living mice (Zhu et al., 2021). In PDT, cytotoxic reactive oxygen materials and singlet oxygen are produced, which results in cell death (Maharjan and Bhattarai, 2022). Materials with a high photothermal conversion efficiency can raise the temperature of the cancerous sites and thereby cause necrosis when used in PPT-based treatments. These are cutting-edge cancer treatment strategies with great promise, and the substances used in these therapies are actively being studied (Bian et al., 2021). Targeting cancerous sites is a natural method to eliminate cancer. By utilizing enhanced permeability and retention effect (EPR) and active targeting, such as modified NPs or dendrimers, they can reach cancerous sites where they release drugs (Kianfar, 2021). Additionally, biomaterials antibodies that target overexpressed particular antigens on the surfaces of cancer cells are widely used (Mercier et al., 2017). Encapsulated drugs are released following endocytosis and either has a cytotoxic effect or nuclear materials cause cell apoptosis, depending on the encapsulated drugs. Successful nucleic acid delivery has been achieved, and extensive exploration and research into transporter-targeted nanoparticulate drug delivery systems based on exosomes (Shtam et al., 2013), liposomes (Landen et al., 2005), dendrimers (Dhumal et al., 2021), and polymeric nanoparticles (Rozema et al., 2007) in cancer therapy (Figure 4).

### 4.1. Targeting cancer metabolism

Cancer metabolism is a potential target for the treatment of cancer in the field of cancer-targeted therapy and nanomedicine. The Warburg effect predicts that they need a significantly higher glucose flux than normal cells because their phenotype is defined by a preference for glycolysis for oxygen-independent energy production (Mullapudi et al., 2020). Due to their essential roles in this aberrant metabolism, several proteins that are overexpressed in cancer have been investigated as potential therapeutic targets. These include phosphoglycerate dehydrogenase, hexokinase-2, and GLUT proteins (Yoshino et al., 2017).

Apart from this, polo-like kinase 1 (PLK1) is overexpressed in various tumors and has been associated with poor clinical outcomes. In addition, PLK1 overexpression has been associated with chemotherapy resistance, and PLK1 inhibition can increase the cancer cell sensitivity to chemotherapy and radiation (Gutteridge et al., 2016; Liu et al., 2017). PLK1 has been the subject of most research as a potential target for cancer treatment. Recently black phosphorus (BP) nanomaterials have been reported to directly affect the centrosome machinery of the cell cycle. BP mechanistically compromises centrosome integrity by causing the deactivation of centrosome kinase PLK1. BP binds directly to PLK1, inducing its aggregation, reducing its cytosolic mobility, and limiting its recruitment to centrosomes for activation. Hence, BP nanomaterials exhibit significant anticancer potential through this mechanism in mice with tumor xenografts (Shao et al., 2021).

In addition, switching out glucose with another monosaccharide like mannose may slow down tumor growth. One promising area for cancer treatment and diagnosis is the repurposing of nanocarriers coupled with glycan-based compounds. Therefore, several different types of functional NPs, such as metalorganic, metallic, and polymeric NPs, are being researched and produced for use in biomedicine (Anniebell and Gopinath, 2018). NPs are small enough to pass through cell membranes and versatile enough to be conjugated with a wide variety of therapeutic and target molecules, which in turn alters their interactions with cell membranes and biological systems, thereby affecting their pharmacokinetic and toxicity profiles (Auría-Soro et al., 2019). It is also possible for the inherent characteristics and dispersion of NPs in living systems to be altered through the conjugation or adsorption of glycoproteins. Since the membrane and milieu of cancer cells have been extensively investigated, multivalent carbohydrates can be used to accomplish binding with glycosylated nanomaterials that interact differentially with tumor-associated glycoprotein receptors (Thomas et al., 2021; Lai et al., 2021).

**Figure 5.**
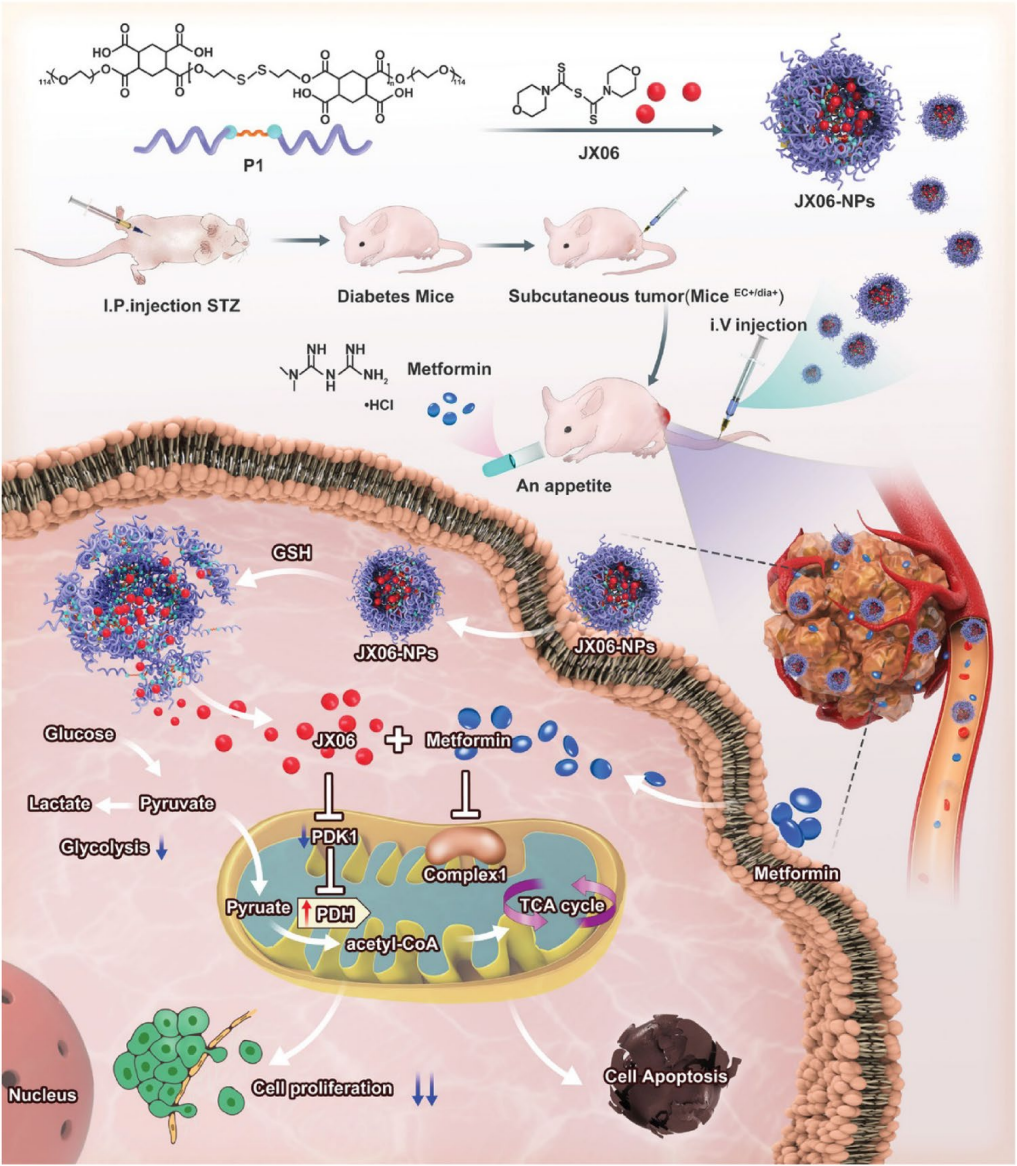
Schematic depiction of JX06-NPs inhibiting the expression of PDK1 in combination with metformin to target EC and modify glucose metabolism for a combinatorial anticancer action. In addition to lowering blood glucose levels, metformin could potentially block oxidative phosphorylation and mitochondrial complex I. JX06-NPs might then release JX06 after being entered into cancer cells, thereby downregulating the PDK1 expression and inhibiting glycolysis. Hence, the combination of JX06-NPs and metformin could expedite EC apoptosis and suppress tumor growth (Yang et al., 2022).

One of the most frequent gynecological cancers is endometrial carcinoma (EC). Metabolic dysfunction and estrogen imbalance play key roles in its pathogenesis. The global prevalence and mortality of EC patients have increased in recent years, paralleling the rising prevalence of metabolic disorders (Siegel et al., 2020; Sheikh et al., 2014). Diabetes has been found to increase the likelihood of developing EC. Diabetic patients have a twofold greater chance of acquiring EC compared to nondiabetic ones. One of the main clinical manifestations of diabetes and a crucial factor in the association between diabetes and cancer is hyperglycemia (Wu et al., 2018). Therefore, a novel strategy for the clinical management of EC patients with diabetes may involve the regulation of blood glucose levels or disruption of the molecular signaling system associated with glucose metabolism. attempted to investigate these novel therapeutic targets (Yang et al., 2022). Ishikawa was cultivated with a high concentration of glucose (IshikawaHG), simulating hyperglycemia in EC patients with diabetes. It was observed that IshikawaHG displayed a reprogramming of its metabolism in response to glucose, with decreased oxidative phosphorylation and increased glycolysis. Proteomics revealed that pyruvate dehydrogenase kinase 1 (PDK1) promotes glycolysis in IshikawaHG. Additionally, even though IshikawaHG is resistant to metformin, the combination of JX06, a new PDK1 inhibitor, and metformin dramatically reduces IshikawaHG proliferation. For drug delivery, JX06 is encapsulated within NPs (JX06-NPs) made from a reduction-sensitive biodegradable polymer. When compared to JX06, JX06-NPs have a greater inhibitory effect on the expansion of IshikawaHG and patient-derived EC cells in vitro. Intravenous injection of JX06-NPs caused them to concentrate in the tumor of an EC-bearing mouse with diabetes, and JX06-NPs with Met combination dramatically reduced tumor growth in EC mice with diabetes. The study showed that using JX06-NPs and metformin together showed promise as a new adjuvant therapy for EC patients with diabetes. This is because it targets the cancer’s metabolism and significantly reduces EC growth (Figure 5).

### 4.2. Targeting metabolic heterogeneity in cancer

Recent applications of advanced technologies like functional genomics, metabolic flux analysis, and metabolomics lend credence to the idea that tumors and cancer cells exhibit distinct metabolic dependencies and preferences. Tumor metabolic phenotypes are highly variable and diverse, with their origins in both the cancer cell itself (through factors such as somatically acquired mutations, differentiation status, and cell lineage) and the surrounding microenvironment (e.g. interactions with stromal cells, interactions with the extracellular matrix, and nutrient milieu). Knowing the proportional impact of these factors on the tumor is essential for understanding the emergence and development of metabolic abnormalities (Kim and DeBerardinis, 2019). Cancer metabolism consists of divergent and convergent metabolic phenotypes. These characteristics provide an understanding of the molecular basis of metabolic reprogramming and its potential therapeutic applications. Many different types of tumors respond in similar ways to a variety of stimuli. These traits include oncogenic pathways that increase redox homeostasis, macromolecule synthesis, and energy production. Malignancy is characterized by a set of convergent features. Convergent properties include glycolysis, which can be triggered by a wide variety of oncogenic triggers and even by hypoxia (DeBerardinis and Chandel, 2016). Divergent properties arise when specific factors activate heterogeneous pathways, resulting in subtype-selective or idiosyncratic phenotypes. The increase of (R)-2HG in cancers with IDH1 or IDH2 mutations and metabolic preferences that distinguish cancers with unique oncogenotypes are examples of divergent pathways (Shackelford et al., 2013). Understanding metabolic heterogeneity is crucial because it affects how we consider utilizing metabolic reprogramming to treat cancer. Divergent features are restricted to subgroups of tumors; therefore, targeting them may result in more tolerable toxicity profiles, but efficacy would be restricted to specific subsets.

Currently, there is no effective treatment available for pancreatic cancer (Rosen et al., 2021). The presence of two types of cancer cell populations in pancreatic cancer is a major challenge in treating pancreatic cancer. It requires targeting each population with a different inhibitor of metabolism for efficient control of the tumor. Proliferating cells utilize glutamine, and their proliferation can be effectively targeted by inducing layered double hydroxide (LDH) NPs containing inhibitors for glutamine metabolism. Slowly dividing cells, called hypoxic cells, are involved in the metabolism of glucose. Metformin, a diabetes treatment drug, can effectively target them. Combination therapy will be based on drugs that can efficiently inhibit glucose and glutamine metabolism (Xu et al., 2022). Although it is a novel therapeutic approach, the available drugs do not ensure optimal efficacy and safety. Elgogary et al. reported the bis-2-(5-phenylacetamido-1, 2, 4-thiadiazol-2-yl) ethyl sulfide (BPTES), a selective and insoluble glutaminase enzyme inhibitor, encapsulated in PLGA-PEG NPs for combinational therapy of pancreatic cancer (Elgogary et al., 2016). BPTES NPs exhibit enhanced pharmacokinetics and efficiency compared to BPTES without encapsulation. CB-839 is an inhibitor of the glutaminase enzyme that is currently being used in clinical trials. It affects the plasma levels of liver enzymes, whereas there is no such side effect of BPTES NPs. A mouse model was used for the investigation in which a patient-derived pancreatic tumor was transplanted. In these models, monotherapy of BPTES NPs exhibits significant antitumor effects. When HypoxCR reporter was used in vivo, it was found that inhibition of glutaminase reduced the growth of the tumor by targeting proliferating cancer cells. However, hypoxic non-cycling cells were unaffected. Metabolomics analysis exhibits that those cells that survived after inhibition of the glutaminase enzyme were dependent on glycogen synthesis and glycolysis. Keeping in view these findings, combination therapy was designed using metformin as a selective drug candidate with BPTES nanoparticles. It resulted in a reduction of pancreatic cancer at a very significant rate compared to the treatment alone. Hence, targeting various cancer metabolic pathways, such as glucose metabolism by nanoparticles-based drug delivery, provides a novel therapy for treating pancreatic cancer.

### 4.3. Suppression of ATP in cancer metabolism

Normal mammalian cells get ATP by mitochondrial oxidative phosphorylation (OXPHOS) and glycolysis. Cancer cells get ATP mostly by glycolysis instead of OXPHOS despite having sufficient oxygen (Vander Heiden et al., 2009). On the other hand, mitochondrial respiration is enhanced in breast cancer cells, glioblastoma, and lung cancer cells when the TCA cycle is triggered, indicating that mitochondrial activity is not necessarily impaired in cancer cells (Bonnet et al., 2007). However, the majority of cancer cells are dependent on glycolysis, which accounts for approximately 60% of total ATP synthesis (Busk et al., 2008). For each glucose molecule, glycolysis generates 2 ATP molecules, making it less efficient than OXPHOS which is capable of generating 36 ATP molecules per glucose. Conversely, glycolysis can produce ATP more quickly than OXPHOS. Cancer cells benefit from enhanced glycolysis because it promotes their rapid growth and multiplication. However, cancer cells upregulate the glycolytic pathway to supply glycolytic intermediates, which are essential for the production of lipids, proteins, and nucleic acids, and for the formation of lactate to maintain the NAD+/NADH redox balance (Sheppard et al., 2021). Therefore, cancer cells get numerous survival benefits when glycolysis is increased rather than OXPHOS.

**Figure 6.**
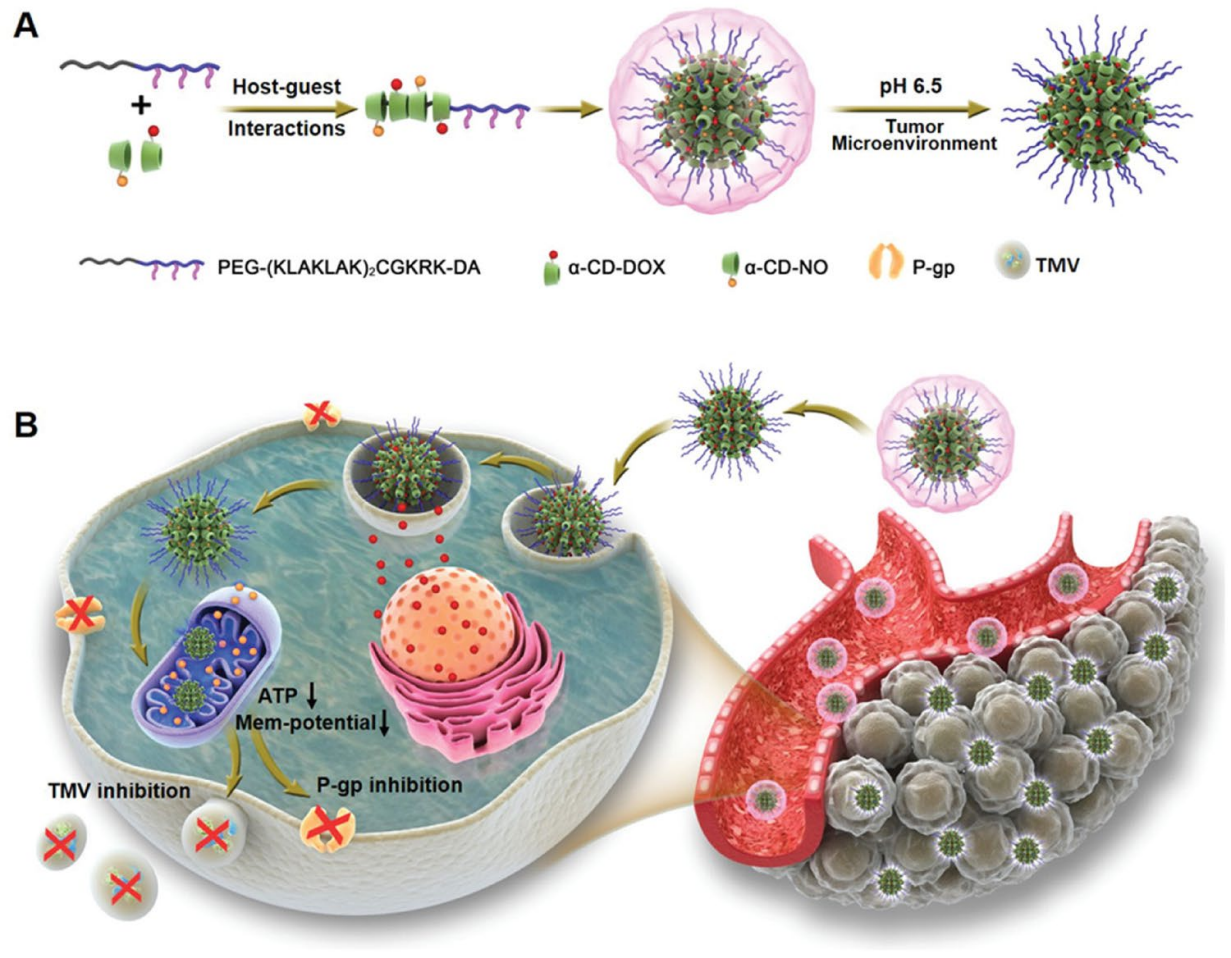
To counteract the effects of medication resistance and cancer spread, this diagram shows how CD-DOX-NO-DA NPs are synthesized and manufactured. A) A schematic representation of the CD-DOX-NO-DA NPs manufacturing process. B) A schematic representation of CD-DOX-NO-DA NPs delivering NO to mitochondria (Deng et al., 2020).

In cancer progression, mitochondria have essential roles in initiation, development, spreading, and other tumor-related processes (Wang et al., 2018). As an example, medication resistance is a significant obstacle in cancer treatment. It is primarily caused by the overexpression of P-glycoprotein and other ABC transporters on cancer cell membranes. These transporters can continuously pump out the chemotherapeutical agents in an energy-dependent manner, resulting in low intracellular drug accumulation and severely reduced cytotoxicity in cancer therapy (Chen et al., 2016). Deng et al. developed an acid-activated mitochondria-targeted nanocarrier for smooth nitric oxide delivery to overcome drug resistance and inhibition of metastasis (Deng et al., 2020). Nitric oxide has the role of suppressing ATP in cancer metabolism for enhanced therapeutic efficiency. The developed nanocarrier was based on α-cyclodextrin, and acid-cleavable dimethylmaleic anhydride modified PEG conjugated mitochondria-targeting peptide. The developed nanosystem increased the blood circulation time and cellular uptake while selectively restoring mitochondria-targeting capability in tumor extracellular pH 6.5. This type of mitochondria-targeted NO delivery is critical in inducing mitochondrial dysfunction by facilitating the membrane permeabilization of mitochondria and decreasing ATP levels, which can inhibit the bioactivities associated with P-glycoprotein and the formation of tumor-derived microvesicles, thereby combating cancer metastasis and drug resistance. Therefore, these nitric oxide delivery NPs that target acid-activated mitochondria are hypothesized to be effective against malignant tumors and may focus attention on other nitric oxide-related cancer treatments (Figure 6).

### 4.4. Targeting mTOR signaling pathway

**Figure 7.**
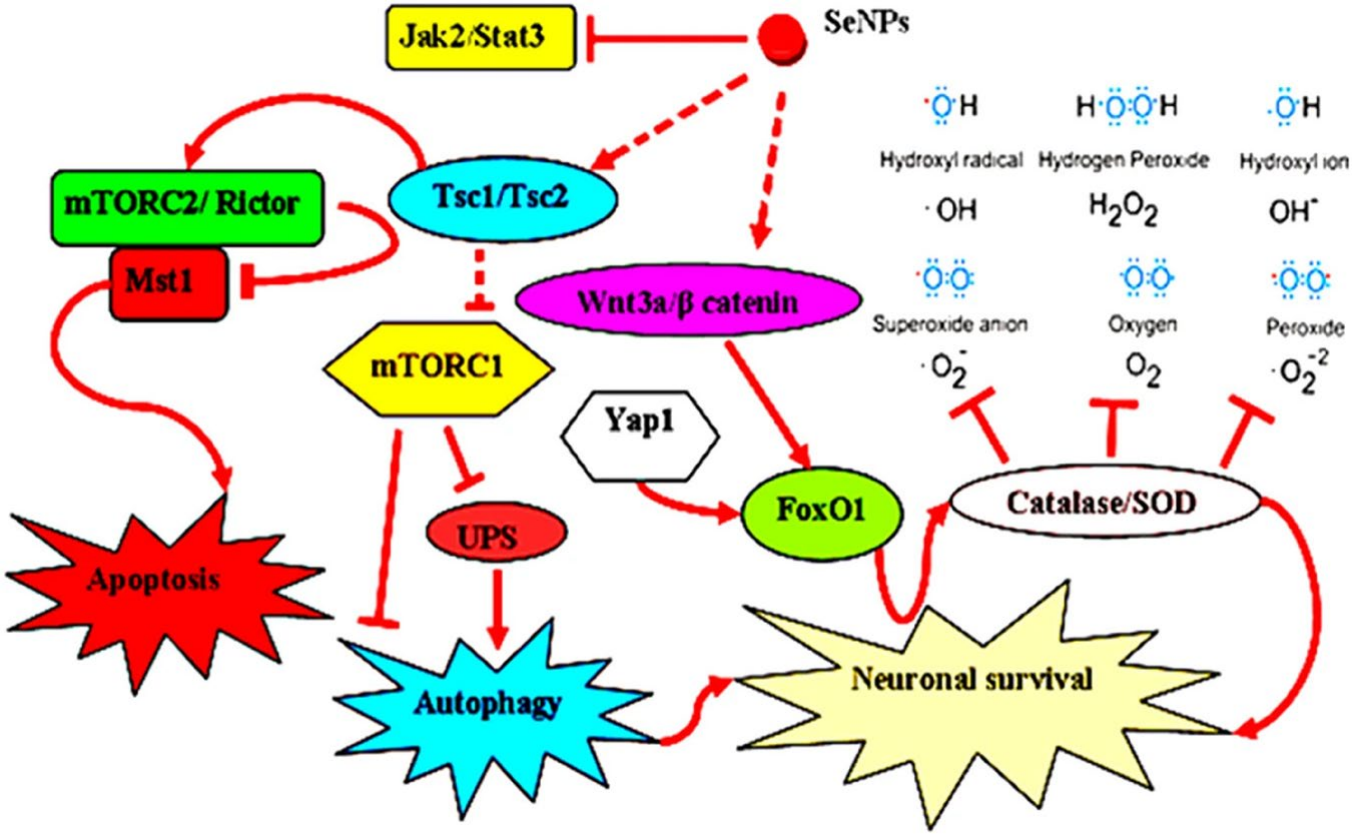
Proposed signaling pathways that are involved in the protection afforded by SeNPs (Amani et al., 2019).

In normal circumstances, a key kinase, serine/threonine kinase mammalian target of rapamycin (mTOR), regulates and controls the growth and proliferation of cells. Various cancers have been found to have mutated mTOR overexpression and some mTOR kinase signaling targets (Meric-Bernstam and Gonzalez-Angulo, 2009). The identification of mTOR signaling as a potential target for cancer therapy. Compared to conventional anticancer medications, mTOR targeting inhibitors have more effective and practical pharmacological profiles. These inhibitors are additionally well tolerated. It is significant to note that several NPs have demonstrated a promising capacity to alter mTOR activity (Lunova et al., 2019). Steady inhibition of mTOR activity by amino-decorated NPs and proliferation in leukemia cell lines can be taken as examples (Mendes et al., 2016). Inhibition of mTOR signaling to be used as therapy has shown limited success. For multiple types of cancers, NPs-based mTOR targeted therapies put forward potential therapeutic choices (Amani et al., 2019). There are challenges and opportunities parallel to progress in the biomedical applications of NPs. Modulation of the mTOR activity is critically analyzed, the complexity of cellular responses to functional NPs is demonstrated, and underlying challenges in identifying the molecular mechanism of the mTOR signaling pathway (Deng et al., 2020). The study provided suggestions for potential cancer therapeutic targets in the near future. It is suggested that the use of mTORC1 signaling in combination with sub-cytotoxic doses of NPs may be used to induce subcellular structural and metabolic changes (Liu and Tang, 2020). The therapeutic effects of NPs on mTOR can be used to modify it, and this knowledge can be used to develop safe and efficient NPs-based cancer metabolism therapies (Figure 7).

### 4.5. Targeting hedgehog signaling pathway

Hedgehog (Hh) signaling has been shown to play a crucial role in human cancer. Currently, smoothened (SMO) inhibitors have been the primary drug targets for studying Hh signaling. Patients with basal cell carcinoma have unfortunately already shown resistance to the currently employed SMO inhibitors (Ramelyte et al., 2022). Therefore, Hh inhibitors that work further downstream in the signaling cascade from SMO may be a more effective strategy. In addition to the classical canonical activation of Hh signaling, non-canonical activation of the GLI transcription factors by multiple important signaling pathways such as TGF, PI3K, and MAPK, highlights the significance of targeting the transcription factors GLI1/2 (Pietrobono et al., 2019). In this context, the most promising agent is likely the GLI1/2 inhibitor GANT61, which has been studied preclinically in numerous tumor types over the past few years (Dusek and Hadden, 2021). GANT61 appears to be highly effective against human cancer cells and in xenograft mouse models, targeting nearly all of the classical hallmarks of cancer, and therefore could be a promising treatment option for human cancer.

**Figure 8.**
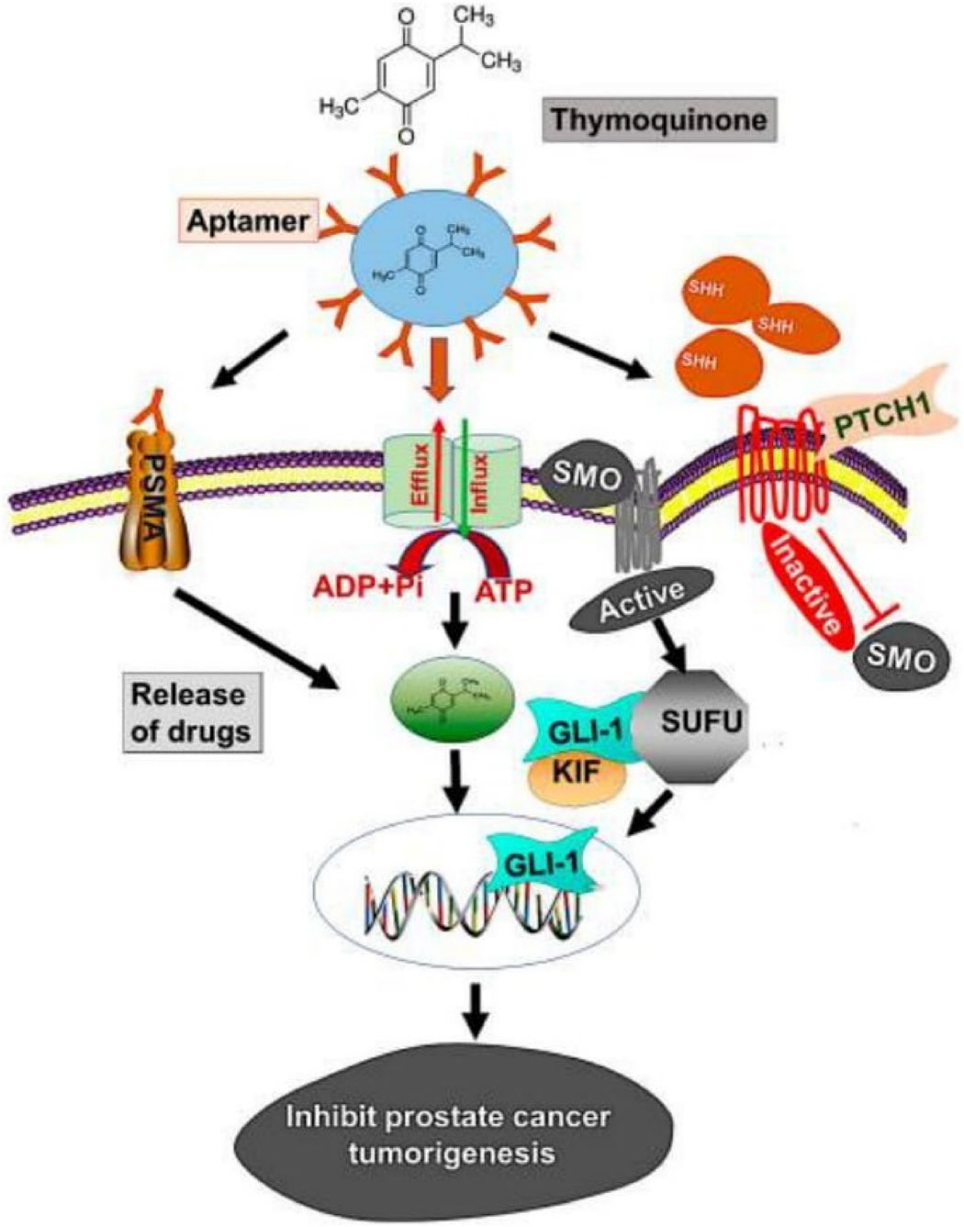
The model demonstrates how PSMA aptamer coupled PBM-NPs can suppress prostate cancer carcinogenesis by targeting the Hh pathway. Regulating the transcription of the ABC transporter gene, the Hh pathway produces chemoresistance. In addition, the Hh pathway involves the activation of SHH, which binds to the receptor PTCH1, followed by inhibition of SMO and, finally, stimulation of GLI-dependent transcription. The suppressor of fused (SUFU) is a transcription factor that binds to the GLIs (GLI-1, -2, and -3) and regulates the expression of their downstream proteins. Modeling the binding of thymoquinone encapsulated A10 aptamer-conjugated PBM-NPs to the PSMA receptor, expressed on the cell surface, revealed the method by which it may be achieved. The aptamer-conjugated nanoparticles blocked the ABC drug efflux protein after being taken up by the cell via endocytosis and transported inside (MDR1). PBM-NPs inhibit cell growth and modify other features of PCa cells by binding to the SHH protein (Singh et al., 2020).

The abnormal Hh signaling pathway is involved in the development of prostate cancer at the molecular level (Hassounah et al., 2012). It causes a more severe case of prostate cancer that is also drug resistant. In normal cells, Hh is involved in regulating major cellular processes, including growth, differentiation, development, and many others. It also has a role in tissue regeneration and repair. Prostate cancer (PCa) in humans exhibited overexpression of Hh pathway molecules, glioma-associated oncogene homolog 1 (GLI-1), and sonic hedgehog (SHH). Thymoquinone is a natural compound and has a significant effect in controlling Hh signaling in prostate cancer. Singh et al., reported planetary ball-milled nanoparticles (PBM-NPs) composed of a natural polysaccharide and carrying in thymoquinone coated with an A10 RNA aptamer (Singh et al., 2020). The function of this aptamer is to bind with prostate-specific antigen membrane antigen (PSMA). The PBM-NPs showed selective targeting of the Hs signaling pathway in docetaxel-resistant PCa. The study revealed that NPs based strategy has the potential to suppress the Hh signaling pathway and hence suppress the progression of PCa (Figure 8).

### 4.6. Targeting reactive oxygen species or oxidative stress

Relatively high levels of pro-oxidants (such as reactive oxygen species (ROS), reactive nitrogen species (RNS), free radicals, and reactive lipid species) in relation to antioxidant capacity are referred to as ‘oxidative stress.’ (Sies, 2015). Elevated levels of ROS are produced by cancer cells, which are essential for both the initiation and the progression of tumors. Compared to normal cells, cancer cells require more energy for growth and invasion, necessitating metabolic expansion and reprogramming to maintain subcellular functions. Regarding this, evidence suggests that cancer cells alter their sugar, sulfur-containing amino acid, and lipid metabolism (Prather et al., 2019). In addition, mesenchymal stromal cells can contribute to the oxidative phosphorylation (OXPHOS) capacity of cancer cells by transferring mitochondria or mitochondrial DNA to cancer cells (Mohammadalipour et al., 2020). Thus, cancer cells produce more ROS, which promotes cancer through increased redox signaling, disruption of the electron transport chain, and mutation of mitochondrial DNA (Prather et al., 2019). Cancer cells can transfer their oxidative stress to cancer-associated fibroblasts (CAFs), stimulating autophagy and supplying high-energic nutrients to CAFs like lactate or ketones that are reported to boost mitochondrial metabolism (Feissner et al., 2009). Thus, tumor cells maintain a high number of functional mitochondria by connecting mitochondrial biogenesis and mitophagy. Furthermore, these cells produce intercellular tunneling nanotubes (TNTs) that facilitate intracellular mitochondrial transport, resulting in the formation of non-cancerous cells in the TME (Lisanti et al., 2010). Tumor cells produce GSH to keep redox homeostasis and prevent ROS-mediated cell death, as ROS causes lipid and protein oxidation and the accumulation of cytotoxic metabolites (Benfeitas et al., 2017). Cancer-specific redox alters metabolic events that promote carcinogenesis.

It has long been believed that suppressing oxidative stress in cancer cells is a promising therapeutic strategy, and the antioxidant’s role in supporting the immune function of the cell cannot be overstated. The safety of antioxidants used as adjuvant therapy in cancer treatment protocols is still under debate because ROS are involved in the mechanism of action of common cytotoxic drugs (Khurana et al., 2018; Thyagarajan and Sahu, 2018). However, recent studies suggest that ROS production could serve as an effective anticancer therapy (Afshari et al., 2019; Jalili-Nik et al., 2020). Important cell signaling cascades, including some that have been linked to cancer cell damage, are regulated by oxidative stress. In this regard, various studies have linked NPs-induced oxidative stress to cancer cell apoptosis (Horie and Tabei, 2021).

Medicines containing metals may alter biological responses by causing an increase in ROS. Transition metals have been shown to promote free radical generation and lipid peroxidation (LPO), potentially leading to oxidative injury of unsaturated fatty acids, decreased membrane fluidity, and which ultimately induced cell death (Chang et al., 2021). For transition metals to be valuable and efficient therapeutic agents, they must selectively destroy diseased cells via oxidative radicals. Recently, a novel green AgNPs synthesized by Nostoc Bahar M (N-AgNPs) was synthesized and investigated for their antitumor effectiveness against various cancer cells (e.g. HepG2, MCF-7, and HCT-116). AgNPs significantly increased lactate dehydrogenase leakage, increased oxidative stress by affecting antioxidants, and induced metabolic tension by significantly lowering ATPase levels; all of these had significant effects. The stimulation of caspase 3 and p53, as well as the suppression of the mTOR pathway via the downregulation of apoptosis-evading proteins, caused apoptosis in HCT-116, MCF-7, and HepG2 cells. It has been established through ultrastructural, biochemical, and molecular studies that N-AgNPs increase apoptosis in all cells studied by either inducing oxidative stress or directly interfering with cellular networks. Therefore, Nostoc-mediated AgNPs represent a promising cancer therapeutic strategy due to their ability to simultaneously target multiple cell death pathways (Hamida et al., 2020). In another study, the anticancer activity of polyethylene glycol (PEG)-stabilized, RNase A-linked gold nanoparticles (AuNPs) against colorectal cancer cells (SW-480). The developed nano-biomedicine AuNPs-PEG-RNase A was found to be effective in inducing apoptosis in SW-480 cells, leading to a significant reduction in the viability of cancer cells. Furthermore, the highest level of ROS generation was observed after 24 hours of treatment with an IC50 dosage of AuNPs-PEG-RNase A, indicating the potential of nanosystem to be considered as a treatment for colorectal cancer (Khiavi et al., 2020). Alginate hydrogel co-loaded with cisplatin and AuNPs was proposed as a multifunctional nano-platform in an exciting new study. After being exposed to the nanoplatform, KB cells showed a 4.4-fold increase in intracellular ROS levels compared to their naive counterparts. In the gene expression analysis, the pro-apoptotic component Bax was found to be upregulated by 4.5-fold, while the anti-apoptotic component Bcl-2 was downregulated by 0.3-fold (Alamzadeh et al., 2020).

Thus, ROS-mediated therapies based on ROS-generating agents, such as photodynamic therapy (PDT) (Yu et al., 2018) and chemodynamic therapy (CDT) (Lin et al., 2018; Lin et al., 2019), are being developed to disrupt cellular self-adaptation mechanisms and induce cell death. The PDT utilities light-activated photosensitizers capable of converting oxygen (O_2_) to ROS (Lucky et al., 2015), whereas CDT takes advantage of an in situ Fenton or Fenton-like reaction between hydrogen peroxide (H_2_O_2_) and catalysts to generate cytotoxic hydroxyl radical (•OH) (Tang et al., 2019). Increased oxidative stress and a greater PDT effect in 4T1 malignant cells were achieved through the use of polymer-encapsulated carbonized hemin NPs (P-CHNPs). When compared to traditional PDT, P-CHNPs-based PDT has been shown to reduce tumor size in xenografted mice. Interestingly, the study showed that P-CHNPs are biocompatible and did not affect hematological and biochemical markers or vital organs (Lin et al., 2022). During PDT, Fe_3_O_4_ NPs can induce the Fenton reaction in tumor cells, converting hydrogen peroxide to hydroxyl radicals. It is worth noting that hydrogen peroxide is the more stable and common ROS molecule produced in large quantities in the cancer cell, and providing excess ferric ions to cancer cells enhances Ros generation that was hindered by hypoxic TME (Tran et al., 2022). In this regard, when exposed to conventional PDT (chlorin E6 (Ce6, a photosensitizer) plus NIR irradiation), U87 MG cells showed inadequate therapeutic responses; however, after using Ce6-loaded manganese ferrite NPs, all malignant cells were eradicated due to increased ROS production and decreased hypoxia (Kim et al., 2017).

**Figure 9.**
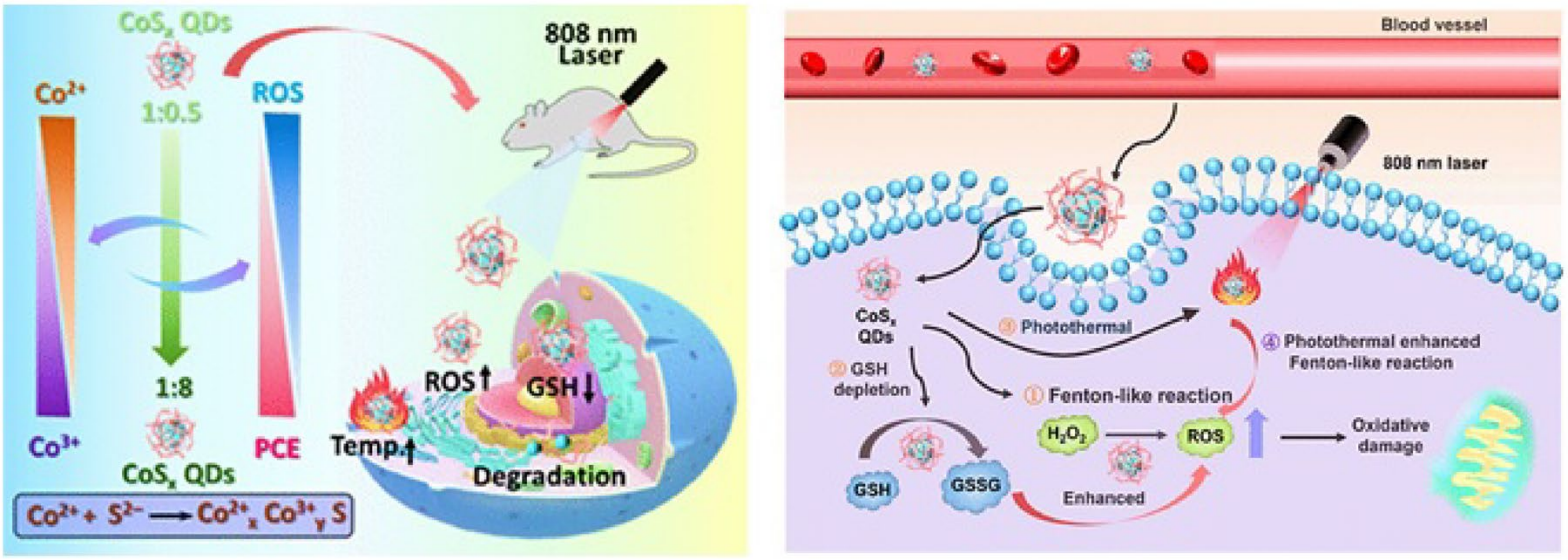
Schematic Illustration of Biodegradable CoS*_x_* QDs for PTT and Hyperthermal-Enhanced CDT of Tumors. Reproduced with permission from ACS 2022 (Zhu et al., 2022).

Recently, it has been widely investigated that combining PDT and CDT can increase tumor oxidative stress and produce a better anticancer therapeutic effect than monotherapy (Liu et al., 2019; Liu et al., 2018). To better synergize photothermal and hyperthermal enhanced CDT of tumors, a series of sulfur-deficient engineered biodegradable cobalt sulfide quantum dots (CoSx QDs) were formed to regulate the photothermal conversion efficiency (PCE) and Fenton-like activity (Zhu et al., 2022). Researchers modulated the PCE and enhanced the Fenton catalytic capability of CoSx QDs through defect engineering. As the number of defect sites increased, the Fenton-like activity increased and produced more toxic •OH, while the photothermal effect decreased slightly. In addition, the QDs’ ultrasmall size and biodegradability enabled them to be rapidly degraded to ions that were easily excreted after therapy, thereby lowering biogenic accumulation and systemic adverse effects. Photothermal and hyperthermal enhancement of the chemodynamic effect of CoSx QDs can assist impressive anticancer activity with favorable biocompatibility, as shown by in vitro and in vivo results. The defect-driven mechanism for the photothermal enhanced Fenton-like reaction in this study provides a flexible strategy for dealing with different treatment environments, holding great promise for the future development of a multifunctional platform for cancer treatment (Figure 9 and Table 1).

**Table 1.** Examples of various NPs and their cancer therapeutic potential in targeting cancer metabolism.

Nanosystems	Drug/Inhibitor	Cancer Type/Cell Line	Key findings	References
Black phosphorus (BP) nanomaterials	–	Hella Cell and A549 cells	BP directly binds to PLK1, aggregating it, decreasing its cytosolic mobility, and restricting its centrosome activation. This mechanism gives BP nanomaterials great anticancer potential in tumor xenografted mice	(Shao et al., 2021)
Reduction-sensitive polymer NPs	JX06, a novel PDK1 inhibitor And Metformin	Endometrial Cancer	In a high-glucose setting, the synergistic effect of JX06-NPs and Met was more pronounced, suggesting a novel approach to the treatment of patients^EC+/dia+^.	(Yang et al., 2022)
Polymeric NPs based on block copolymer of PLGA)and PEG.	bis-2-(5-phenylacetamido-1,2,4-thiadiazol-2-yl)ethyl sulfide (BPTES), a glutaminase inhibitor.	Pancreatic cancer	Combination therapy of Metformin with BPTES nanoparticles significantly reduced the pancreatic tumor more than either treatment alone	(Elgogary et al., 2016)
An acid-activated mitochondria-targeted drug nanocarrier	Doxorubicin	4T1 breast cancer cells	Mitochondrial-targeted NO delivery effectively inhibits TMV formation by cutting off the vital energy supply, demonstrating excellent antimetastatic capability in an orthotopic 4T1 tumor model	(Deng et al., 2020)
*Nostoc*-mediated SNPs	–	MCF-7 cells, HCT-116 cells, and HepG2 cells,	N-SNPs induced apoptosis in MCF-7, HCT-116, and HepG2 cells by activating caspase 3 and p53 and suppressing the mTOR signaling pathway by downregulating apoptosis-evading proteins	(Hamida et al., 2020)
Polyethylene glycol (PEG)-stabilized AuNPs	–	Colorectal cancer cells (SW-480)	AuNPs-PEG-RNase A significantly decreased the viability of SW-480 cancer cells by inducing apoptosis. In addition, the ROS production assay results supported the ability of the engineered nanomedicine to induce ROS production, highlighting the potential of this system in cancer therapy via the ROS-induced mediated pathway	(Khiavi et al., 2020)
Alginate hydrogel co-loaded with cisplatin and gold nanoparticles	Cisplatin	KB human mouth epidermal carcinoma cells.	Tri-modal therapy increased anticancer efficacy by elevating intracellular ROS levels in KB cells by a factor of 4.4 compared to untreated cells	(Alamzadeh et al., 2020)
Carbonized hemin nanoparticles		4T1 malignant cells	Carbonized hemin nanoparticles exhibited excellent in vitro and in vivo PDT effects owing to improved ROS generation efficiency, glutathione depletion, and hypoxia relief.	(Lin et al., 2022)
Manganese ferrite nanoparticle-anchored mesoporous silica nanoparticles	Chlorin e6 (Ce6), photosensitizer	Human glioblastoma cancer (U-87 MG) cells	MFMSNs relieve hypoxic conditions using a small number of nanoparticles and improve therapeutic outcomes of PDT for tumors in vivo by utilizing the continuous O2-evolving property of MnFe2O4 nanoparticles via the Fenton reaction	(Kim et al., 2017)
Cobalt sulfide quantum dots (CoSx QDs)	–	4T1 cells	CoSx QD produced sufficient ROS for cell inactivation without the addition of H_2_O_2_ via a photothermally-enhanced Fenton-like reaction.	(Zhu et al., 2022)

## 5. Challenges and future perspectives

Nanotechnology has shown promising results in delivering small molecules for cancer detection, diagnosis, therapy, and circumventing MDR. Nanoparticles provide opportunities for improved therapeutic outcomes for small molecules and biologicals due to their submicron-sized colloidal particle size and targeting capacity (Sakamoto et al., 2010). However, there remain many unanswered questions and challenges associated with their clinical development, stability, and toxicity.

Nanoparticle development is challenging due to the lack of suitable *in vitro* models capable of mimicking accurately the *in vivo* state. Current therapeutic applications of nanoformulations are based on *in vitro* evaluation using cell lines which fail to capture the complexity of nanoparticle-cell interactions *in vivo* (Valencia et al., 2020). Preliminary *in vivo* performance and toxicity testing of nanoparticles using suitable animal models must be carried out carefully to identify and overcome the risks associated with and minimize the adverse effects of nanotechnology on human health and the environment. This is an emerging area and it remains unknown as to which class of nanoparticles provides the best approach to treating cancer.

In the development of NPs-based delivery and targeting, three major factors should be considered: efficacy enhancement, side effect minimization, and resistance avoidance. In many instances, the NPs based delivery and targeting may simultaneously address multiple problems because of its instinct mechanism. However, surface modification of NPs with specific targeting ligands makes them a superior delivery vehicle and often demonstrates significantly higher transfections effectiveness than nonmodified ones (Khot et al., 2021).

Research regarding NPs-based delivery and targeting in cancer treatment is expanding rapidly, but many issues remain unresolved. One of the major concerns about nanoparticles remains their potential toxicity. Physiological barriers are breached due to the nanoscopic size, which may offer health risks (Gimeno-Benito et al., 2021). There is evidence that free radicals generated by NPs damage DNA, organelles, and biological membranes (Xia et al., 2006). By interacting with cell surface receptors, NPs supplied intracellularly may trigger an immune response. Since the toxicity of NPs is dependent on a wide variety of circumstances, they must be modified during production to minimize their hazardous effects.

Another major challenge in the application of NPs for cancer treatment is the mononuclear phagocytic system (MPS) (Xiao and Gao, 2018). NPs adsorb proteins to produce plasma cells. These plasma cells target MPS for the uptake of MPS. Designing of NPs that target macrophages can be used as an alternative with proper research and experimental studies. Currently, strategies being used are inhibiting macrophage recruitment, reprogramming and depleting tumor-associated macrophages (TAMs), and obstructing CD47-SIRPα pathways (Adityan et al., 2020).

Study-design challenges are significantly contributing to the failure of successful NPs-based clinical trials (Anselmo and Mitragotri, 2016). Some of the study-design challenges include the size and timing of certain NPs therapies. Most of the experimental studies of NPs are based on cell and animal models. It may not provide convincing results in human trials. In addition, as metastasis is a significant property of cancer, there is a need for proper research on models of cancer metastasis (Guimarães et al., 2018). Genetic, past medical history and environmental factors need to be considered while designing NP-based cancer therapies.

The combination of the valuable potential of nanotechnology with the unique capabilities of the human immune system can be very promising for cancer treatment. Combining the benefits of nanotechnology with other promising methods holds great promise for the development of effective and innovative cancer therapy tools. The convergence of nanomedicine with immunotherapy, cancer cells, and genes is anticipated to transform the future of cancer therapy. In addition, the unique optical properties of NPs in conjunction with the high specificity and affinity of certain biomolecules, such as aptamers and antibodies, have the potential to create highly sensitive and efficient biosensors for early cancer detection. Combining these approaches for the design of smart nanostructures that can identify and image tumors, along with multiple and targeted cancer therapies, could be a breakthrough in cancer treatment.

## 4. Conclusions

Cancers have become a formidable obstacle for cancer researchers due to their high mortality rate and resistance to therapeutic procedures. Multidrug resistance, recurrence, and the spreading nature of cancer cells, for instance, make cancer extremely difficult to treat. Nanotechnology is an emerging field that has contributed to different fields of research and technology. Cancer researchers have broadly investigated the potential of various NPs in cancer treatment. NPs-based multiple therapies have been investigated and studied for cancer therapy. Numerous strategies have been applied, studied, and analyzed, such as encapsulating drugs with NPs, targeting glucose metabolism in tumor cells, enzyme-responsible for cancer metabolism, and many others. All these cancer-treating strategies came out with promising results and opened the gates of potential future perspectives regarding cancer therapies. Studies have shown that NPs-based cancer therapies ensure more efficiency in drug delivery, efficacy, and stability. Moreover, NPs-based treatments are readily available and affordable, with minimal side effects in the long run. Several nanomedicines are approved by the Food and Drugs Authority (FDA) that are successfully turning the lives of cancer patients back to life. Still, much work has to be done to make NP-based cancer therapies efficient in clinical use. Most of the studies are being conducted and successful in animal models like mice. The promising results motivate cancer researchers to further explore different types of NPs, their cancer therapeutic potential in targeting cancer metabolism, challenges, and ways to overcome them.

## Ethics approval and consent to participate

This does not involve any animals or humans.

## Consent for publication

All authors approved the final version of the manuscript.

## Data Availability

Not applicable.
